# 肺癌图表演化见证转化性研究发展

**DOI:** 10.3779/j.issn.1009-3419.2016.06.21

**Published:** 2016-06-20

**Authors:** 潮 张, 文昭 钟

**Affiliations:** 510080 广州，广东省肺癌研究所，广东省人民医院肿瘤外科 Department of Surgical Oncology, Guangdong Lung Cancer Institute, Guangdong General Hospital and Guangdong Academy of Medical Sciences, Guangzhou 510080, China

**Keywords:** 肺肿瘤, 转化性研究, 免疫治疗, Lung neoplasms, Translational research, Immunotherapy

## Abstract

肺癌从传统化疗到分子靶向，再到如今免疫治疗的转变，转化性研究发挥着无可替代的作用，其中图表演化更是见证一次次重大变迁，从“森林图”到“生存曲线图”，“瀑布图”，“蜘蛛图”再到最近的“时间线区域面积图”，纵向展示了肺癌治疗从群体逐渐向个体深入细化的理念和演进过程。尽管目前最新的免疫治疗炙手可热，但其研究结果并没有达到预期理想，同时传统的治疗手段仍然存在局限性，需要更深入探索。本文将从图表演化角度论述肺癌转化性研究的发展历程，剖析部分失败的外科临床研究，以期对未来肺癌治疗及图表演化有所启发。

## Abstract (en)

Lung cancer treatment has altered from conventional chemotherapy to targeted treatment, which now has been turned to the immunotherapy. Translational research has played an irreplaceable role during this progression which graphic evolution has witnessed. The evolution has gone through forest plot, KM-curve, waterfall plot, spider plot and timeline-area, showing us the refining concept and gradual process of lung cancer treatment undergoing from community towards individual. Even though the latest immunotherapy is getting increasingly hot, the result isn't quite expected. Meanwhile, the limitations of conventional treatment still exist which require further research. This article will primarily illustrate the development of translational research of lung cancer via the aspect of curve evolution and analysis some abortive clinical trials in lung cancer surgery for inspiring the next graphic style and lung cancer treatment.

在过去的数十年间，肺癌转化性研究在推动肺癌治疗前进的道路上起了举足轻重的作用，从传统化疗时代到靶向治疗时代，再到如今炙手可热的免疫治疗时代，无处没有转化性研究的身影，而图表演化更是如影随形（图 1），从“森林图”，“生存曲线图”到“瀑布图”和“蜘蛛图”，再到“时间线区域图”，每一次图表的演化和流行都见证了肺癌治疗的进步。目前的传统化疗以及靶向治疗在肺癌临床应用上都已日渐成熟，尤其是靶向治疗，在不断探索新的治疗性靶点的同时，也在不断深化旧靶点的新治疗模式，然而肿瘤耐药一直是困扰靶向药物发展的瓶颈。而免疫治疗的出现为我们打开了肺癌治疗的另一扇大门，目前对免疫治疗的探索仍处于初级阶段，是否存在人种差异、最合理的靶点选择，相关的研究数据仍有待更大样本前瞻性试验验证。本文将从图表演化角度论述肺癌转化性研究的发展，以期对未来肺癌治疗及图表演化有所启示。

**1:**
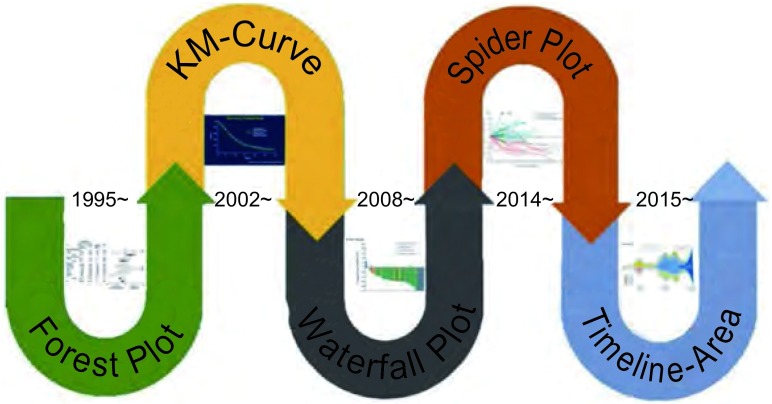
肺癌转化性研究的图表演化 Sketch map of graphic evolution in translational lung cancer

## 1 转化医学

转化医学最初由“实验室到临床”这一概念演变而来，Geraghty^[1]^于1996年在*Lancet*杂志第一次提出，并将其描述为基础科学新发现与临床实践的“联姻”。到2003年鉴于高昂的研究支出与不相符合的群体健康水平及医疗现状，美国国立卫生研究院（National Institutes of Health, NIH）正式提出转化医学，并全面阐述了这一概念，强调基础研究成果需转化为有效的临床治疗手段^[2]^，转化医学也愈来愈受到广泛关注，其中肺癌存在最丰富的治疗性驱动靶点，因而转化研究尤为引人瞩目。肺癌作为全世界癌症的头号死因之一，尽管诊疗技术日新月异，其5年生存期仅15%，数据显示每年全球肺癌死亡人数超过100万人，关于肺癌转化性研究的脚步从未止步^[3, 4]^。

## 2 肺癌转化性研究的发展

### 2.1 “森林图”传统化疗时代（1995年-）

1995年在*British Medical Journal*上发表的一篇基于52项随机对照试验（randomized control trial, RCT）研究，共计9, 387例患者的荟萃分析无疑为非小细胞肺癌（non-small cell lung cancer, NSCLC）化疗无效时代画上了休止符^[5]^。尽管此前已有化疗应用于肺癌的先例，但主要用于发病率只有20%左右的小细胞肺癌（small cell lung cancer, SCLC），而在80%的NSCLC（包括鳞癌和腺癌）患者中，仍没有确切有效的药物。该研究结果显示无论是联合放化疗和单纯放疗的比较，或支持治疗联合化疗和单纯支持治疗的比较，均可以明显提高其2年/5年生存期，并降低死亡风险，随后关于各种化疗药物和化疗模式在肺癌治疗中的应用也在此荟萃分析基础之上被不断发掘和延伸，可以说*British Medical Journal*这篇荟萃分析是现代肺癌临床研究的开山之作。而这股肺癌化疗的荟萃分析浪潮也在席卷中国，大量荟萃分析如雨后春笋般层出不穷。随后逐步确立了含铂双药在晚期NSCLC中的一线治疗地位，时至今日在分子靶向及免疫治疗的双重“夹击”下，化疗在晚期NSCLC的一线治疗仍占据着重要地位。其中“森林图”的应用更是以其直观的死亡风险对比结果“预见”了化疗在肺癌治疗中的一次重大变迁，同样也包括目前随机对照临床试验中众多的亚组分析。

### 2.2 “生存曲线图”靶向治疗时代（2002年-）

尽管肺癌化疗在很长一段时间内处于一枝独秀的地位，2002年一篇发表在*New England Journal of Medicine*关于4种第三代化疗方案在晚期NSCLC中比较的多中心RCT无疑让我们看到了肺癌化疗的“平台期”，4种第三代化疗药之间并未显示出明显临床疗效差异^[6]^。随后2003年首个分子靶向药物吉非替尼（Gefitinib）面世，肺癌治疗掀开了崭新的一页，但这种肺癌分子靶向指导临床治疗的方法仍处于起步阶段^[7]^。2005年ISEL研究遭遇滑铁卢，这个RCT对比的是表皮生长因子受体酪氨酸激酶抑制剂（epidermal growth factor receptor-tyrosine kinase inhibitor, EGFR-TKI）吉非替尼（Gefitinib）和安慰剂在不经选择的复治性NSCLC的疗效和预后，虽然最终阴性结果发表在*Lancet*杂志，使通过快速通道进入美国食品药品监督管理局的吉非替尼（Gefitinib）被撤离，患者和整条产业链也因此而蒙受无法估量的损失，但从这份严谨数据背后的亚组分析发现，某些特殊类型的人群，如亚裔、非吸烟的腺癌可从该药物中明显获益，促使日本和美国科学家的基础临床转化性研究揭示这一亚群患者有表皮生长因子受体（epidermal growth factor receptor, EGFR）活化突变率高的特征，从而一步一步的揭开了个体化分子靶向治疗的序幕^[8, 9]^。随后的IPASS研究更是一石激起千层浪^[10]^，从4条经典的生存曲线中，我们能很清晰地看到对在临床选择人群非吸烟腺癌和EGFR阳性患者使用吉非替尼可以获得无进展生存期（progression free survival, PFS）的明显提高，而在EGFR阴性组中却出现截然相反的结果。然而IPASS之所能成为靶向治疗的里程碑，不仅仅只是在于它提示了*EGFR*突变在指导EGFR-TKI的临床应用，更是给我们提出了这种基于靶点的靶向治疗思路，为今后靶向药物研发及应用起到了至关重要的作用；另一方面IPASS也让我们认识到即使靶向药物在非选择人群中有效率很低，但并不意味着这种靶向药物毫无价值，如果经过合理的靶向患者筛选，有效率可能可以得到明显的提高，同时也对我们的临床试验设计提出了进一步的指导意义，更深化了靶向富集试验的重要意义。在此之后，靶向药物得到了飞速地发展，无论是OPTIMAL试验抑或Lux-Lung6试验，都让我们惊喜地看到了生存曲线的大幅度“跨越”，而OPTIMAL更是首次将晚期NSCLC患者的中位无进展生存期延长至1年以上^[11]^。之后关于肺癌治疗的研究也开始从追求药物临床获益向治疗模式的探索转变。抗血管生成联合含铂双药化疗（贝伐珠单抗联合卡铂和紫杉醇）可作为非鳞NSCLC患者标准一线方案（BEYOND），尤其是EGFR状态未明或野生型患者。和十年前同样设计的ECOG 4599相比，生存期翻倍，生存曲线形态迥异，归因于亚裔和欧美人种的遗传特征差异和近十多年肺癌领域治疗的飞跃。此外在IPASS和针对腺癌的CheckMate057研究都有共同的生存曲线交叉特征，提示免疫治疗潜在的分子预测标记物。

在此期间，肺癌外科临床研究领域也进行了很多探索，主要集中在围术期治疗、肺实质和淋巴结切除范围、分期和外科技术革新等几个重要方向。在2015年第16届世界肺癌大会（World Conference on Lung Cancer, WCLC）发布的重要研究E1505，这项对比术后辅助化疗联合或不联合贝伐单抗，为期十年，样本量达1, 501例的Ⅲ期临床研究结果，今年美国临床肿瘤学会（American Society of Clinical Oncology, ASCO）上再次被列为口头发言。与其说是因为这项研究改变了术后辅助治疗的临床实践，倒不如说E1505壮烈的阴性结果给辅助治疗领域研究模式带来的深刻反思。2004年后术后辅助治疗这些年遭遇的滑铁卢，从BR19到RADIANT，失败者众。和进展期肺癌临床研究中位4.3年的完成时间相比，目前开展的肺癌外科相关临床试验普遍周期较长，从临床研究的生命周期图（图 2）中我们可以看到，截至2013年止，肺癌外科临床试验中位完成时间9.4年，而新辅助/辅助治疗相关试验则要超过10年。由于高度的个体化，随访时间长，外科医生工作繁忙，外科临床研究，是一个艰难的领域，肺癌研究进展日新月异，外科和辅助治疗的结果需要漫长的等待时间，今天的研究设计难以预测到十年后的状态。经典例子莫过于日本开展的肺叶对比亚肺叶Ⅲ期试验（JCOG0802/WJOG4607L）^[12]^，该研究在LCSG821阴性结果基础上，调整了试验设计，弥补了入组标准的不足，扩大纳入样本量，尽管2014年才入组1, 038例患者，仍未达到计划入组量，但作为一个大样本前瞻性随机对照研究，期待能在下一个五年甚至十年内看到具有重大变革意义的结果。此外淋巴结清扫相关试验，ACOSOG开展的一项Z0030研究同样跨度十年^[13]^。该研究为关于淋巴结清扫范围的多中心，随机对照的前瞻性研究，旨在比较完全性纵隔淋巴结清扫和纵隔淋巴结采样在T1N0/非肺门N1的NSCLC患者中的生存获益。尽管该研究结果显示两者间无明显生存差异，但该研究得出完全性淋巴结清扫可以为NSCLC提供精准分期。

**2:**
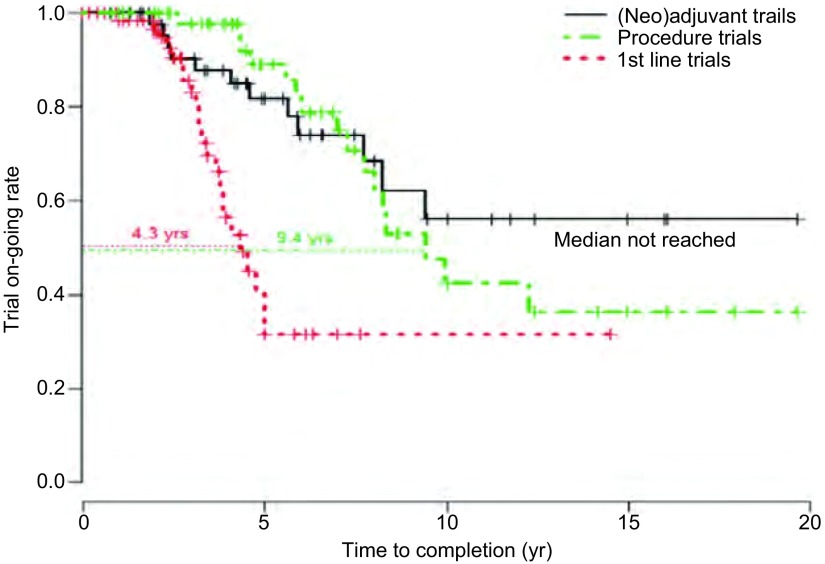
Ⅲ期肺癌外科临床试验完成时间 Time to completion of phase Ⅲ clinical trial lung cancer surgery

近期在*Journey of Clinical Oncology*上刊登了国内七大医学中心开展的回顾性研究，该研究依据多个独立预后因素按权重评分构建了临床列线图（nomogram），以指导临床医师更精准评估NSCLC术后患者的生存期，为不同亚组患者提供个体化的治疗策略，该研究在原有的TNM分期基础之上更细化了分组及预测标准，更加体现了当今精准化治疗理念。然而目前由于部分回顾性数据分析存在的固有缺陷，未来于真实世界数据的前瞻性研究具有更高效广泛的应用前景^[14, 15]^。

### 2.3 “瀑布图”罕见突变时代（2008年-）

总生存期作为诠释临床试验结果的“金标准”，延用了数十年之久，而Lewis的一项关于尝试将肿瘤体积变化作为研究终点的试验中^[16]^，首次对肿瘤体积能否作为治疗相关预测因子进行验证，尽管结果仍不能明确其作为预测因子的可行性，但不可否认肿瘤体积变化情况对反映治疗是否有效具有早期提示作用。另一方面，分子靶向药物在治疗上似乎并不能使所有接受治疗患者肿瘤缩小，推动了肺癌转化性研究从更具群体化的“森林图”及“生存曲线图”向更能反应“小众群体”的“瀑布图”演进。其中Van Allen EM等^[17]^的一项基于511例病例的前瞻性队列研究表明大部分癌症患者存在着不止一个驱动基因的突变，提示传统的临床试验设计方法可能并不能满足于多驱动基因突变肺癌个体的研究。而这也对后续相关肺癌转化性研究的革新提出了更高的要求，篮子试验和雨伞试验也相继应运而生，其中雨伞试验更是将这种罕见突变事件集中起来，变少见事件为“常见事件”，无论是在加速罕见突变疾病的临床试验还是对于个体获得精准治疗上，都具有重大意义。而后续的研究中，一篇关于纳武单抗（Nivolumab）与多西他赛（Docetaxol）在晚期肺鳞癌中的比较的前瞻性随机研究（CheckMate017）^[18]^让我们初略了“瀑布图”的变种。CheckMate017作为一项针对一线化疗失败后肺鳞癌的Ⅲ期临床试验，具有里程碑式意义，其结果相当令人鼓舞，在持续治疗过程中，其中位总生存期（overall survival, OS）较对照组整整提高了3个月，1年生存率更是高达42%，同时还让我们看到了在肺鳞癌中纳武单抗（Nivolumab）的疗效与细胞程序性死亡配体1（programmed death-ligand 1, PD-L1）的表达水平无关，为临床治疗决策提供参考。之后在2015年欧洲癌症大会（European Cancer Congress, ECC）上更是报道了一篇“重量级”研究，关于Rova-T（rovalpituzumab-tesirine）用于化疗耐药后晚期SCLC的Ⅰ期临床试验，研究结果显示DLL-3（Delta-like 3）高表达的患者，其应答率较对照组有明显提高，而今年ASCO上更是有力压联合免疫治疗的出色表现，让我们看到了SCLC靶向治疗的潜在可能性。

### 2.4 “蜘蛛图”免疫治疗时代（2013年-）

免疫治疗这一概念早在19世纪中期就已出现萌芽，然而受限于当时的研究技术等问题，无法很好将其转化到相关疾病的治疗中，直到2011年随着美国食品药品监督管理局批准了易普利姆玛（Ipilimumab）上市，免疫治疗开始逐步走上正轨。鉴于免疫治疗药物临床疗效出现缓慢，传统评价实体瘤标准（Response Evaluation Criteria in Solid Tumor, RECIST）无法全面地体现免疫治疗疗效，免疫治疗疗效评价标准（immune-related response criteria, irRC）应运而生。相较于传统RECIST而言，irRC以直径5 mm作为新发病灶是否纳入肿瘤负荷分界点，排除了一些可能由于免疫细胞浸润而导致在影像学上观察到的假阳性结果，但目前irRC仍存在一定局限性，如免疫治疗疾病稳定（immune-related stable disease, irSD）无法很好区分是细微的肿瘤负荷变化还是快速进展的肿瘤负荷随之降低到基线处这两种情况，因此仍需要更进一步优化相关标准定义及更深入的研究^[19]^。相比于“瀑布图”，“蜘蛛图”不仅提供了更多患者相关信息以及应答情况的数据，同时能容纳更庞大的样本人群，最重要的是可以动态反映个体肿瘤变化情况，迎合了个体免疫治疗中肿瘤定期监测以及反应不同个体治疗应答情况的观察需求。虽然一直以来晚期肺癌被认为是免疫抑制性环境及免疫原性低甚至缺失而致使其对免疫治疗应答较差，但在2012年一项关于评估抗PD-L1抗体应用于晚期癌症免疫治疗安全性及有效性的试验中^[20]^，该研究结果表明应用抗PD-L1抗体的晚期肺癌患者存在较多个体可以维持肿瘤长期缩小的状态且延长病情稳定期。另外我们知道晚期的肺鳞癌一般预后较差，而且一线铂类药物治疗的后续治疗选择有限，CheckMate017和CheckMate057的研究让我们看到了纳武单抗（Nivolumab）在鳞癌和非鳞NSCLC患者接受含铂化疗后的治疗中，能带来明显生存获益，而且随着随访时间的延长可以获得持续的生存获益^[18, 21]^。

### 2.5 “时间线区域面积图”克隆丰度演化（2014年-）

随着二代测序技术（next-generation sequencing, NGS）和液体活检的发展，我们看到了更多可操作的靶点基因，然而在分子靶向治疗相关临床试验中，分子靶向药物耐药问题也日趋明显，如NSCLC经典靶点EGFR，尽管EGFR-TKI的临床应用给EGFR阳性肺癌患者带来绝处逢生的希冀，但好景不长，对TKI初始治疗有效的患者最终都难逃耐药及治疗失败的命运。如果说“蜘蛛图”单纯呈现了分子靶向中个体化的肿瘤大小变化，那么“时间线区域图”无疑将导致分子靶向治疗药物耐药的深层原因揭示出来。在Alice的研究中发现原本治疗前的ALK C1156Y亚克隆在经过克唑替尼（Crizotinib）治疗后，该亚克隆扩增到50%，并导致了肿瘤的发生进展^[22]^，然而在应用了对ALK C1156Y这种亚克隆敏感的Lorlatinib后，该克隆获得二次ALK突变ALK L1198F，这种双基因突变亚克隆又对Lorlatinib不敏感，导致肿瘤耐药的再次发生，然而空间构象改变使得该肿瘤细胞又再次对克唑替尼敏感。“时间线区域面积图”呈现了ALK耐药与癌细胞此消彼长，此起彼伏的动态变化过程。

## 3 展望

从肺癌的转化性研究发展历程来看，我们不难发现每一次肺癌治疗阶段的前进总会伴随着图表的演化，从群体的“森林图”，“生存曲线图”到小众的“瀑布图”“蜘蛛图”，再到个体的“时间线区域面积图”，每一次的递进都让我们看到了肺癌治疗手段或治疗模式的变革。传统靶向治疗一大阻力是易诱导获得性耐药，而免疫治疗作为后起之秀，目前并没有像靶向治疗那样找到具有潜在临床获益的适合人群，在今后肺癌治疗中仍有很大的潜在提升空间。随着更多免疫治疗相关的深入研究，我们有理由相信在不久的将来势必会掀起另一场肺癌治疗变革的风暴，届时又将会有怎样新的图表演化来展现见证这一新治疗成果呢？我们拭目以待。
